# Skydiving: The audiological perspective

**DOI:** 10.4102/sajcd.v65i1.553

**Published:** 2018-05-28

**Authors:** Dhanashree Pillay, Shaaista Dada

**Affiliations:** 1 Department of Speech and Hearing Therapy, School of Human and Community Development, University of the Witwatersrand, South Africa

## Abstract

**Background:**

Skydiving is a popular recreational sport for the young and old. There is minimal research pertaining to skydiving and its relation to the audiological system. The risks of skydiving in relation to the auditory system should be explored further.

**Aims:**

The main aim of this study was to explore the relationship between skydiving and audiology in South Africa. The sub-aims of the study focused on determining if skydivers were provided with safety precautions before they commenced with the dive, determining the middle ear pressure before and after the skydive and identifying the audiological symptoms that were present post-dive. This study also aimed at scrutinising the South African sports and recreation policy.

**Method:**

A mixed-method descriptive research design was utilised. Qualitative information pertaining to audiology was identified and recorded from the scrutiny of South Africa (SA) policy and the dropzone consent forms at two skydiving schools. Thirty-one skydivers were purposefully recruited to undergo a pre- and post-dive tympanometric assessment.

**Results:**

There is no information within the clearance forms that pertain to the audiological risks related to skydiving. There was a lack of information related to the risks of skydiving in the clearance forms at both dive schools. A statistically significant pressure change was noted in regular skydivers, regardless of the ability to equalise effectively during the skydive.

**Conclusion:**

This study identified the gaps in policy and clearance forms, highlighting the need for the inclusion of safety measures and risks in the documentation and legislation that governs the sport. Audiologists, sportspeople and medical advisors should be cognisant of the negative consequences that may be evident within the auditory system of skydivers.

## Introduction

Risk-taking behaviours are usually considered to be socially unacceptable and reckless with negative consequences. However, high-risk sports are regarded as socially acceptable because of the thrill and excitement of the sport (Castainer, Le Scanff & Woodman, 2013). Participants of high-risk sports are usually sensation seekers who thrive on the increased stimulation (Zuckerman, 1990). Examples of high-risk extreme sports include rock climbing, skydiving, skiing, scuba diving, white water rafting and bungee jumping.

Serious consequences of extreme sports may include skull fractures, vertebrae fractures, loss of consciousness, barotrauma or even death (Burke, 2013; Gutovitz, Weber, Colern, Papa & Giordano, 2008). Barotrauma occurs because of the lack of pressure equalisation, and it is the most common medical complication arising from diving and aviation (Bentz & Hughes, 2012; Du Plessis, Fothergill, Gertner, Hughes & Schwaller, 2008). Barotrauma to the ear may affect the middle or inner ear with symptoms and signs such as tinnitus, vertigo, otalgia, hearing loss or rupture of the tympanic membrane (Bentz & Hughes, 2012). Alternobaric vertigo may be a consequence of skydiving as this phenomenon is caused by the difference in pressure between the middle ear space and inner ear which consequently causes vertigo (Bentz & Hughes, 2012). Therefore, skydiving is an extreme sport that may negatively affect the hearing mechanism of the participant. The risks involved in participating in a sport should be highlighted in the consent forms that are signed by the participant. This will ensure that the participant is fully aware of the negative consequences that may occur. Sports and recreational legislation should aim to protect the participant in a sport; therefore, safety aspects should be included in the documentation. The *Safety at Sports and Recreational Events Act* of 2010 does not include any information that is related to the safety of sports participants (Republic of South Africa, 2010), and the safety section within the legislation pertains to the attendance of a sporting event.

The sporting associations in South Africa (SA) are responsible for the safety of their participants. Skydiving in SA is governed by the Parachute Association of South Africa, using a Manual of Procedures as approved by the South African Civil Aviation Authority. This manual was last updated in July 2012 and suggests that a sport skydiver should provide his or her chief instructor with a medical certificate which states that he or she is medically fit to skydive (Parachute Association of South Africa [PASA], 2012). The *South African Boxing Act* of 2001 mandates that the Boxing Association of South Africa (BSA) must ensure that the safety of boxers and the relationships between boxers, managers, promoters, trainers and officials and BSA are effectively and efficiently administered and governed in the best interests of boxing and its stakeholders as a whole (Republic of South Africa, 2017).

Recreational scuba divers in SA undergo a medical examination that includes a hearing assessment (Pillay & Jardine, 2010), in light of the failure to adequately equalise ear pressure that may result in a ruptured tympanic membrane and subsequent pain (Strauss & Aksenov, 2004). Scuba diving and skydiving are popular in SA (Visser, 2004) despite the risks of barotraumas being evident in both sports. Recreational skydivers may obtain a license from the Parachute Association of South Africa; however, it is not obligatory (PASA, 2012). The participant is not mandated to have follow-up medicals to determine the effects of frequent jumps on the auditory system. Furthermore, in skydiving, untrained participants are able to strap themselves to a qualified tandem master and experience the sport without a licensing requirement.

A study in Florida, USA, found that the average pre-jump middle ear pressure was −23.5 daPa, the average post-jump pressure was −70.5 daPa and the participants experienced aural fullness, otalgia, vertigo, or a sensation of a loss in hearing abilities (Gutovitz et al., 2008). Based on these symptoms, the long-term consequences of frequent participation in the sport may lead to permanent auditory damage. The higher the altitude of the jump and a longer time of freefall during the jump are factors that may negatively impact the middle ear system of a frequent jumper. This highlights the need for better regulation and awareness among participants about the symptoms.

Internationally and in the South African context, there is a scarcity of research conducted with the participants of the ever popular sport of skydiving. The knowledge gaps pertaining to the relationship between skydiving and audiology are evident in the dearth of literature available. However, the growth in popularity necessitates that further investigations are conducted to ascertain the audiological consequences of participation in the sport. This study was conducted to add to the body of knowledge in SA that relates to the effect of skydiving, specifically on the middle ear system. The popularity of skydiving in SA necessitates the exploration of clearance forms to ensure that the skydiver is aware of the risks. The effects of skydiving on the auditory system need to be documented to inform the skydiving population of the potential risk of the sport.

## Methodology

### Aims and sub-aims

The main aim of this study was to explore the relationship between skydiving and audiology in South Africa.

The sub-aims of the study are as follows:

- to review the dropzone pre-jump consent forms at the skydiving schools and document any information related to audiology
- to determine what policies govern skydiving in SA and document any hearing-related risks and precautions
- to assess the difference in middle ear pressure pre- and post-skydive
- to determine the audiological symptoms experienced post-skydive
- to document the skydivers’ perceptions about the possible ear-related risks when skydiving.

### Research design

A mixed-method descriptive research design was employed in this study. Qualitative information was obtained from reviewing of policies, clearance forms and open-ended questions on a questionnaire. The information extracted from the clearance forms and from the scrutiny of the policies that govern the sport in SA was transcribed onto a recording form. A tympanometry assessment was conducted at 5 min before flight for the jump and immediately after landing post-jump. The quantitative information was obtained from the tympanometry results, specifically the changes in the middle ear pressure, because of the altitudes experienced during skydiving.

### Sample

Thirty-one participants (21 males and 10 females) were recruited using a purposive sampling design. The group was divided into regular (more than five jumps) skydivers (*n* = 13) and first time skydivers (*n* = 18).

Inclusion criteria for participants were as follows:

- 18 years or older as seen in Table 1
- either first time skydiver or an experienced skydiver.

Exclusion criteria were as follows:

- Participants that have had a previous audiological problems such as:
  ■ previous middle ear surgery
  ■ grommets
  ■ recurrent middle ear infections
  ■ otitis externa
  ■ occluding cerumen
  ■ a pre-dive tympanogram that indicated a possible middle ear abnormality.

**TABLE 1:** Age range of participants.

Age range of participants	Number of participants
18–25 years	11
26–35 years	8
36–45 years	7
Above 45 years	5

The above-mentioned exclusion criteria were included as these factors have an impact on the results obtained in tympanometry. A middle ear infection yields negative pressure, whereas grommets increase the volume of the ear canal volume (Gutovitz et al., 2008).

### Data collection procedure

#### Data analysis

The pressure changes during a skydive were the focal aspect of the assessments, and hence, the pressure measured in daPa was documented and analysed using Stata Statistical Software. Pre- and post-tympanometry results were analysed with a paired *t*-test to determine if they were statistically different (*p* < 0.05). Qualitative data from the questionnaire were thematically analysed according to the common themes that emerged.

## Ethical consideration

Ethical clearance was initially obtained from the medical ethics committee; thereafter, two skydiving schools in SA provided the research with permission to access the skydivers. Consent forms were completed by all participants. South African policy documents from the South African Parachute Association (SAPA) were scrutinised. The SAPA is the officiating body that oversees the health and safety aspects with the sport. Clearance forms from the two skydiving schools were obtained, and the audiologically relevant information from the clearance form was recorded on a record form. The second phase included the use of questionnaires to 31 skydivers, and the third phase included a middle ear assessment via tympanometry, pre- and post-skydives.

## Results

### Clearance forms and policies

The information in the clearance forms pertaining to the audiological risks such as barotrauma to the middle or inner ear was non-existent. The clearance forms used are not standardised but self-developed by the skydiving school; therefore, findings cannot be generalised. There was no evidence of any medical clearance requirement for a tandem passenger, which places the first time jumper at risk.

### Pre- and post-skydive tympanometry

There was no statistically significant difference between the left and right ears. When assessing the regular and non-regular skydivers, the pre- to post-jump pressures are statistically significant at the 5% level, and hence both groups reveal a statistically significant difference (Table 2).

**TABLE 2:** Average ear pressure changes.

Skydiver	Pre-dive		Post-dive		Change	
	Mean (daPa)	Confidence intervals	Mean (daPa)	Confidence intervals	Mean (daPa)	Confidence intervals
Regular divers	5.36	(−16.77 to 27.49)	−2.09	(−23.45 to 19.27)	−8.30	(−12.87 to −3.74)
Non-regular divers	−28.00	(−59.85 to 3.85)	−99.05	(−148.95 to −49.16)	−67.11	(101.40 to −32.81)
All Subjects	−14.89	(−35.96 to 6.17)	−60.96	(−96.25 to −25.67)	−42.45	(64.42 to −20.47)

### Audiological symptoms experienced post-skydive

**FIGURE 1:**
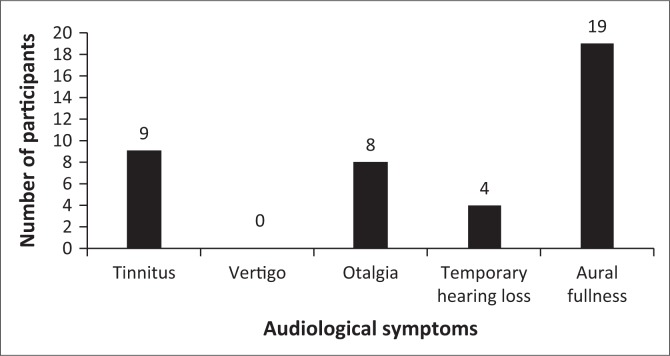
Audiological symptoms experienced by participants.

Nine participants reported having experienced tinnitus after the skydive (Figure 1). The majority of participants in the study reported a sense of aural fullness which was either unilateral or bilateral. There were four participants who reported a temporary threshold shift after the skydive. The researcher did not measure the time taken for the temporary symptom to subside.

### Skydivers’ perceptions about the possible ear-related risks when skydiving

Twenty-two participants reported having an understanding about tympanic membrane rupture and its causes. It was noted that of the 22 participants that understood this phenomenon, 12 of them had a previous tympanic membrane rupture from the sport. Participants highlighted that loud noises may pose a risk to hearing, and one participant noted that ear infections and ‘eardrum’ bursts may be a cause of hearing loss. Only 13 participants reported that they read the dropzone pre-jump consent form before signing it.

Fifteen participants did not foresee any health or audiological risks related to skydiving. It was further noted that five participants saw death as a more likely consequence. Another five participants noted an awareness of possible physical injury as a result of the skydive. Four participants noted an awareness of a possible decrease in hearing over a sustained period of skydiving.

## Discussion

Participants are at risk of ear trauma as there is a lack of information provided to the prospective new skydiver at both skydiving schools. The lack of precautions on the clearance forms places the sportspeople in a vulnerable and dangerous position, as a tympanic membrane perforation can occur at the first jump creating a middle ear pathology and a conductive hearing loss (Bentz & Hughes, 2012). South African Sport and Recreation policies focus on the safety at sports events, whilst specific sporting associations are responsible for the safety of participants. The Parachute Association of South Africa, the governing body that is responsible for the safety measures that are implemented in the sport (PASA, 2012), needs to include more detailed information pertaining to the different options of skydiving and the related risk to the hearing system to ensure that all participants are making informed decisions. The findings of this study indicate that the dropzone consent forms were developed at the discretion of the owners at each school. The lack of an official consent form, with the inclusion of safety rights, is evidence for the development of a standardised form that is endorsed by the PASA.

The number of participants in this study who read the consent forms concurred with participants who partake in other extreme sports in SA, as a study regarding recreational scuba diving (Pillay & Jardine, 2010) ascertained that only 45% of the participants in their study had signed the consent form without being fully aware of the details in these forms.

The symptom of tinnitus can be attributed to the rapid middle ear pressure change (Gutovitz et al., 2008) during the dive or the loud noise experienced from the aircraft during flight pre-dive. The aural fullness experienced is as a direct result of the pressure change that is experienced during the skydive. The findings indicate the need for awareness about the audiological consequences that may result from extreme sports such as skydiving. This study illustrates the need for a larger study that considers how different sports have an effect on the auditory system.

## Conclusion

Skydiving has become a popular sport; however, research regarding the sport and its audiological consequences is minimal. As a result, participants have little to no knowledge of the effect that skydiving has on the auditory system. It was noted that individuals who only participated in the sport once were not educated about the manner and time at which to equalise during the skydive. The results from this study clearly depict a pressure change – which poses a hazard of tympanic membrane perforation and middle ear barotrauma. Although the study provides useful information, there are some limitations that must be considered. It must first be noted that the research only took place at two skydiving schools, and therefore, the generalisability of the results is limited. However, the study draws attention to the risks associated with skydiving. Audiologists, sportspeople and medical advisors should be cognisant of the negative consequences that may be evident within the auditory system of skydivers.
